# Capturing dynamics in nursing: a diary study of nurses’ job characteristics and ability and willingness to continue working

**DOI:** 10.3389/fpsyg.2023.1112530

**Published:** 2023-07-31

**Authors:** Renée Cornelia Josephina Maria Vermeulen, Evelien Hanna van Leeuwen

**Affiliations:** ^1^Utrecht University School of Governance, Utrecht University, Utrecht, Netherlands; ^2^University Medical Center Utrecht, Utrecht, Netherlands

**Keywords:** nursing profession, work experiences, employability, diary study, longitudinal research

## Abstract

**Objectives:**

This study aimed to gain insight into (1) the dynamics of job characteristics (demands and resources) of nurses and (2) how job characteristics relate to nurses’ ability and willingness to continue working (understood as employability). Job characteristics are profession-specific and vary over time, but studies often overlook these dynamics. Moreover, job characteristics relate to nurses’ employability, which is under pressure due to a rapidly changing work environment. It is necessary to gain insight into the dynamic job characteristics of nurses to develop targeted workplace interventions that help nurses remain employable.

**Methods:**

This study adopted a mixed methods diary approach, with a strong emphasis on qualitative analysis. 46 Nurses from two hospitals in a large Dutch city completed a structured diary at six points over 3 weeks, resulting in 225 diary entries in total. The nurses used a custom-made application on their mobile devices to describe in their own words what they experienced as demanding and resourceful throughout the shifts and how they experienced their employability. Prior to the diaries, nurses completed an intake survey.

**Results:**

A within-person analysis highlighted the day-to-day dynamics in the nursing profession. The job characteristics a nurse mentioned in the first diary entry often were completely different from the job characteristics the same nurse mentioned in the other diary entries. This analysis also showed variety within nurses’ employability, demonstrating that perceptions of employability vary over time. A between-person analysis highlighted links between job characteristics and nurses’ employability: social interactions strengthen a nurse’s employability, a strict task approach threatens it, and aspects such as a strong drive to care, professional development, and autonomy (clustered as aspects that enable to “act professionally”) create opportunities to enhance employability.

**Conclusion:**

Insights from this study show that job characteristics typical to the nursing profession can be linked to nurses’ perceptions of employability. Also, it stems from this research that not only job characteristics but also employability are dynamic in the short run. Understanding and enhancing employability in a nursing context requires capturing these dynamics, for instance by collecting data at several points in time or by using mixed-method studies to understand employability scores within their context.

## Highlights

- Longitudinal diary data were collected at six moments in time using a diary approach which enables both within- and between-person comparisons.
- A diary approach is novel and innovative to collect the work experiences of healthcare professionals.
- The understanding of various job characteristics was deepened and nuanced to match the dynamic nature of the nursing profession.
- The study shows that job characteristics as well as employability are dynamic and vary over different points in time.
- The benefits of the longitudinal approach were to an extent offset by some participants withdrawing.

## Introduction

The work environment of nurses is one that never stands still. Both internal and external developments proceed rapidly. It is no longer a question of if, but rather of how, these developments affect nurses’ physical and mental ability as well as their willingness to remain employed in nursing until their retirement age (Van Vuuren et al., 2011; Oude Hengel et al., 2012a), which is defined as employability (Oude Hengel et al., 2012b). Internally, changes in nurses’ work environment demand new competencies and skills to remain able to work (Van Harten, 2016). Externally, the COVID-19 pandemic (Chen et al., 2021) has challenged healthcare workers’ motivation and ability to continue working (Lai et al., 2020; Vindrola-Padros et al., 2020; Van Leeuwen et al., 2021). Furthermore, the healthcare workforce is aging and struggling to recruit and retain younger members (Herkes et al., 2019; De Lange et al., 2020). Specifically for the nursing profession, large studies such as the European Nurses’ Early Exit Study (NEXT; Hasselhorn et al., 2003) have shown that the already substantial shortage of nurses is threatened even more by an increasing number of nurses wanting to leave their jobs (Hasselhorn et al., 2003). This has resulted in fewer nurses being available while the demand for care is increasing. These challenging conditions emphasize the importance of investing in nurses’ employability.

The antecedents of employability have been widely studied and it has been demonstrated that job characteristics affect employability (Nilsson et al., 2011; Oude Hengel et al., 2012a; Van Harten et al., 2022). Job characteristics can be divided into job demands, referring to job aspects that require sustained physical and/or psychological effort or skills and job resources, referring to job aspects that reduce the negative impact of these demanding aspects and help achieve work goals, and stimulate personal growth and development (Demerouti et al., 2001, p. 501). Job demands, such as physical and emotional demands, have been shown to negatively affect employability (Nilsson et al., 2011; Oude Hengel et al., 2012a). Job resources, such as supervisory support, have been shown to increase employability and, conversely, a lack of supervisory support negatively impacts employees’ employability (Oude Hengel et al., 2012a). Related to this, Van Leeuwen (2022) has shown that job demands like workload relate negatively to healthcare professionals’ employability, whereas job resources such as support from colleagues relate positively to their employability. Moreover, a study using the NEXT data explains that their highly demanding work setting limits nurses’ employability (Van der Heijden et al., 2020). Consequently, it is generally assumed that nurses’ work experiences impact whether they are able and willing to continue their work.

Research building on the job demands-resources (JD-R) model (Demerouti et al., 2001; Bakker and Demerouti, 2007; Tims et al., 2012) demonstrates that job characteristics vary between professions (Demerouti et al., 2001; Bakker and Demerouti, 2007). For nurses, the quantitative workload and work-life interference are perceived as demanding aspects of the job while supervisory support, psychological support, and autonomy are important job resources (Broetje et al., 2020). In addition, the NEXT data argues that the extent to which nurses are committed to the organization is mainly affected by the meaning of their work (Van der Heijden et al., 2020). Moreover, research stresses the importance of balancing job demands and job resources to create positive work outcomes. The JD-R model posits that job resources have the potential to buffer the negative effects of job demands on work stress (Schaufeli and Taris, 2013). For example, having autonomy may enable employees to cope with a high workload. However, balancing between job demands and job resources is arguably a challenge as job characteristics can vary weekly and even daily (Bakker and Sanz-Vergel, 2013; Tadić et al., 2015).

Studying job characteristics thus requires explicitly accounting for both the dynamics over time and the professional context. Despite this, a meta-analysis showed that studies into the relationship between job characteristics and job outcomes (such as employee wellbeing) have mainly used cross-sectional or two-and three-wave approaches to collect data (Lesener et al., 2019). Although this has contributed to our understanding of the relationships between job characteristics and job outcomes, using more than 3 measurement moments allows us to better capture the dynamic nature of job characteristics.

Our study contributes to earlier work by showing which job aspects should be viewed as demanding against resourceful for nurses by considering the dynamic character of these profession-specific aspects. These dynamics are especially important in the nursing profession as workdays are typically never the same. The following research question is addressed in this study: *how do dynamic job characteristics* (i.e.*, job demands and job resources*) *relate to nurses’ employability?* With this question, we aim to deepen the understanding of nurses’ job characteristics and to increase insight into how dynamic job characteristics relate to their employability. We use a diary approach to map the dynamics in nurses’ job characteristics as it includes “time” as a factor (Bolger et al., 2003; Chan et al., 2018).

## Methods

### Design and setting

A longitudinal mixed methods diary approach with a strong focus on the qualitative component investigated how the dynamics in nurses’ job characteristics relate to their employability. A diary method fits this study’s research aim as it allows us to capture nurses’ daily experiences of their job demands and resources. While diary approaches are already used to examine patients’ experiences (Anwar et al., 2017; Chan et al., 2018; Webster et al., 2019), this is one of the first studies using a diary approach to examine nurses’ experiences. In addition to the diaries, respondents also completed a survey that serves as an intake tool. The survey collected demographical data of the participating nurses and was designed in Qualtrics. The DearScholar application (hereafter: app; Kruyen, 2020) collected qualitative and quantitative data as it allowed participants to answer questions digitally. The app was custom-programmed to match the design of this research. For an impression of the design and layout of the app, see Appendix 1.

### Participants

Nurses from two hospitals participated in this study. Both these hospitals are public and located in the same large Dutch city. The first hospital has 1,000 beds and the second hospital has 550 beds, and the hospitals employ 1,900 (hospital 1) and 1,100 (hospital 2) nurses, respectively. Four nurses were involved in the pilot test of the study: two were working as nurses in the first hospital, and two were working as a nurse in the second hospital. All nurses were female, and the nurses represented different age groups. The nurses provided feedback on the content, relevance, wording, and layout of the app. Based on the pilot, the wording and layout of the app were slightly adjusted. For instance, we changed the personal pronouns to make the tone less formal (in Dutch: je/jij instead of u), and we changed some icons to better match the content.

For the main study, a maximal variation sample strategy was used. All nurses working at these two hospitals were invited to participate in the study. Nurses who worked as a nurse at the time of the data collection were included in the study. The Nursing Advisory Council or the Works Council sent one e-mail with the invitation to all its nurses. This invitation consisted of an introductory message about the study. It highlighted that the researchers wanted to know more about nurses’ daily work experiences in relation to their job satisfaction. The message was accompanied by a flyer which also included information on the topic and method of this study, as well as the starting date and duration of the study. Lastly, the invitation provided nurses with the contact details of the researchers. Nurses who signed up received an e-mail with the planning and guidelines, including a manual for downloading and using the DearScholar app that is used to collect the data digitally. The study was reviewed and approved by Facultaire Ethische Toetsingscommissie (FETC) of the Faculty of Law, Economics and Governance (LEG). Participating nurses had to provide written informed consent stating that participation was voluntary, outcomes were confidential, all study material would be anonymized, and participants could withdraw at any time. Nurses who provided their written informed consent were included in the study. In total, 46 nurses participated in this diary study. An overview of the participating nurses and their characteristics can be found in Appendix 2. While dropouts are considered inevitable in diary studies (Ohly et al., 2010), 26 nurses (56.5%) completed all six diaries, and 225 diary entries were obtained (see Table 1). This sample size is consistent with other diary studies (De Vries et al., 2019; Bernards et al., 2021).

**Table 1:** Summary of diary entries.

Number of diaries completed	Number of nurses	Number of diary entries
6 diaries	*n* = 26	156
5 diaries	*n* = 4	20
4 diaries	*n* = 7	28
3 diaries	*n* = 5	15
2 diaries	*n* = 2	4
1 diary	*n* = 2	2
Total	*n* = 46	225

### Data measurement

As this study focuses on nurses in the Netherlands, the items used to collect the data were formulated in the Dutch language, and we use our own translations in this paper.

#### Employability

Employability was measured *quantitatively* by assessing nurses’ ability and willingness to continue working in their current profession until retirement age (Oude Hengel et al., 2012a; Van Leeuwen et al., 2021a). We used three items based on the work of Oude Hengel and colleagues: “I feel [physically (item 1)/ mentally (item 2)] able to remain employed in nursing until the retirement age” and “I am willing to remain employed in nursing until the retirement age” (item 3). These items are frequently used to measure employability in the Dutch context, for example in the Dutch Survey on Work Conditions (NEA; Van Leeuwen et al., 2021b).

#### Job characteristics

Nurses’ job characteristics were measured *qualitatively* through open questions inspired by the Job Demands-Resources model (Demerouti et al., 2001). Respondents reflected on four categories: (a) what gave energy during the shift (job resources), (b) what costed energy during the shift (job demands), (c) what helped them during the shift (challenging job characteristics), and (d) what hindered them during the shift (hindering job characteristics). There was no maximum word count for the responses, and the respondents were free to name everything that came to their minds in the app.

### Data collection

The data was collected in the aftermath of COVID-19. It had two phases, where we collected both qualitative and quantitative data. In the first phase, respondents took an intake survey. The survey included demographical questions and questions about nurses’ workplace and employment conditions. The items and nurses’ demographics are included in Appendix 3. On completing this initial questionnaire, the respondents received an e-mail containing their login code for the DearScholar app.

The second phase consisted of the diary entries using the DearScholar app (Kruyen, 2020). Nurses had to complete six diaries at the end of shifts, with an interval of 3 days between the diary entries. Each entry was scheduled for a different day of the week to capture potential variability in work experiences between shifts. The diary questions concerned their job characteristics and employability. There was an additional option in the app where respondents could ask the researchers questions, give feedback or elaborate on things they considered important. Nurses received two push notifications each day a diary entry was scheduled, reminding nurses to complete the diary. They received the first notification at the beginning of the day saying: “Do you keep in mind to fill out your diary today? Thanks!.” The second notification arrived at the end of the day and said: “Thank you for filling out your diary today!.” On average, respondents took 5 min to complete a diary entry.

### Data analyses

Diary entries were qualitatively transcribed and clustered per respondent to gain an overview on the within-person level. This overview contained information on each respondent’s perceptions of their employability and their experiences of job characteristics at each of the diary entry points. Subsequently, these data were coded in NVivo v.12 (QSR International Pty Ltd, 2018). First, the quantitative scores on employability were qualitatively coded. The mean scores for each component determined whether nurses were able, or not, and willing, or not. A nurse’s employability was coded as structurally positive when the mean score was 3 or above, and negative when the mean score was below 3. Many diary entries by a single respondent showed different scores on employability over time and so we assessed whether the scores for each component of employability were stable or dynamic. A nurse’s employability was coded as stable provided at least two out of three components were stable across the diary entries, otherwise as dynamic. Table 2 provides an example of how employability was coded for one respondent (nurse 29).

**Table 2:** Example of qualitatively coding the quantitative data on employability of nurse 29, whose employability is coded as (a) structurally negative (neither able nor willing), and (b) dynamic.

	Physical ability	Mental ability	Willingness	Employability (total)
T1	Disagree (2)	Neutral (3)	Disagree (2)	
T2	Neutral (3)	Neutral (3)	Disagree (2)	
T3	Neutral (3)	Neutral (3)	Disagree (2)	
T4	Disagree (2)	Disagree (2)	Disagree (2)	
T5	Disagree (2)	Disagree (2)	Disagree (2)	
T6	Fully disagree (1)	Fully disagree (1)	Fully disagree (1)	
Total (a)	Not able (*M* = 2.16)	Not able (*M* = 2.33)	Not willing (*M* = 1.83)	Negative (*M* = 2.11)
Total (b)	Dynamic	Dynamic	Dynamic	Dynamic

Second, the job characteristics were coded using a thematic analytical approach (Braun and Clarke, 2006). This analytical approach allows both deductive and inductive analyses by systematically identifying theory-initiated themes while considering their meaning within the context (Loffe and Yardley, 2004; Vaismoradi et al., 2013). The initial codes generated were relevant to the job characteristics of the nurses in our sample. These initial codes were then collated into potential sub-themes and themes, which were checked and refined using theoretical insights on job demands and resources (Demerouti et al., 2001; Bakker and Demerouti, 2007) as well as knowledge gained in previous studies on job characteristics that were important in nursing (Broetje et al., 2020). An example of this coding strategy is displayed in Table 3. All other codes are included in Appendix 4. Both authors were equally involved in the analytical process as they discussed most of the data entries between them.

**Table 3:** Example of thematic coding of job characteristics.

Quote	Initial code	Sub-theme	Theme
“What stimulated me today was… having a laugh with patients”	Laughing with patients	Social interaction with patients	Interaction with patients
“Today I gained energy from… learning new things”	Learning on the job	Learning and developing	Professional development

## Results

This section reports on the diary entries of nurses at different points across six data entry points in 3 weeks. Over these points, nurses mentioned a great variety of characteristics that were energizing, stimulating, draining, or hindering. In general, nurses described their shifts using a different set of job characteristics in each diary entry, implying that their experiences vary across shifts. This is illustrated by nurse 31 who, on day one, perceived her collaboration with colleagues as energizing, whereas in her second entry it was social interaction with patients that energized her, and in the fourth it was the experience of control and the ability to do something extra. This resulted in a large and broad set of job characteristics. Notably, nurses generally mentioned “only” two or three job characteristics in each diary entry. While each shift could probably be characterized by significantly more characteristics, nurses apparently recollected only a few characteristics and we assumed these were the most prominent for them in that shift.

Alongside the variety in job characteristics across the diary entries, the data also reveal that nurses’ notion of their employability differs over time. Table 4 shows the mean scores for the nurses’ employability at each diary entry. This shows that perceived employability not only differs between nurses but also within nurses. The perceived employability of several nurses shows significant fluctuations as illustrated by nurse 23 who perceived their employability as slightly negative based on the first diary entry, but very negative in the second diary entry, very positive in the third, negative in the fourth, positive in the fifth, and very negative again in the sixth diary entry. There were also nurses whose employability grew more positive over time (e.g., nurses 11 and 26). For a few nurses, the opposite was true: their perceived employability decreased over time (e.g., nurses 29 and 38). Although our study found that employability varied over a relatively short period, our data did not enable explanations for why this is the case. Nevertheless, the great variety within both the employability and job characteristics suggests that the longitudinal diary approach adopted fits well with the dynamic context of nurses. The next subsections probe in more detail which job characteristics may relate to nurses’ employability.

**Table 4:** Mean scores and standard deviations of employability perceptions over time.

	Physical ability		Mental ability		Willingness	
	*M*	*SD*	*M*	*SD*	*M*	*SD*
T1 (*n* = 46)	3.08	1.00	2.70	0.97	2.59	1.00
T2 (*n* = 40)	3.26	0.89	2.93	0.92	2.81	0.94
T3 (*n* = 39)	3.41	0.95	3.00	1.10	2.93	1.19
T4 (*n* = 37)	3.29	0.98	2.87	1.02	2.71	1.11
T5 (*n* = 33)	3.42	0.94	3.06	1.12	2.76	1.03
T6 (*n* = 30)	3.31	1.06	2.81	1.03	2.56	1.05

### The strength of social interactions

The data show the importance of social interactions with four different actors in nurses’ work: patients, colleagues, students, and doctors. Especially nurses who, on average, perceived their employability positively mentioned that social interactions energized and stimulated them and, in so doing, referred to four categories. First, these nurses gain energy from interacting with patients and value having time for conversations and humor (nurses 8 and 17) and are stimulated by patients showing gratitude and connecting with them (nurses 4 and 11). Second, these nurses gain energy from their interactions with immediate colleagues. They appear energized by having fun with colleagues (nurses 6 and 37), by being complimented (nurses 24 and 27) and by giving and receiving help from colleagues (nurses 28, 42, and 29). Third, of those nurses who perceived their employability structurally positive (meaning they perceived their employability positive on average) and were involved in teaching students, they were also energized through their interactions with students, valuing students’ enthusiasm (nurses 39 and 42) and helping them to develop in the nursing profession (nurse 35). Fourth, interactions with doctors may also provide nurses with energy. They value personal contact with doctors (nurse 5) and especially direct contact with doctors as this allows them to make swift decisions (nurses 3 and 43).

Notably, the data show that many of the aspects linked to social interactions require sufficient time as a condition. That is, nurses mentioned that they value the time they have for social interactions in their work, particularly to catch up with colleagues (nurse 15) and interact with patients (nurses 42 and 10). One nurse explained:

*“It is nice to have some time in between work to talk to my colleagues”—*nurse 41.

The data show that inadequate time for social interaction can drain nurses. This lack of time can be the result of a high workload. However, nurses can sometimes appreciate a high workload and it seems that this is only draining if it prevents nurses from socially interacting in their work.

As with the nurses who are structurally positive about their employability, social interactions with different actors also appear an important factor for nurses who are structurally negative about their employability. This group of nurses reports that while social interaction in their work can stimulate them and provide energy, social interactions with patients, colleagues and doctors can also drain and hinder them. First, nurses recalled many negative patient characteristics that drain energy, such as patients that are annoying, delirious, demanding, aggressive, opinionated, indecisive, and disordered. Second, the negative work attitudes of some colleagues were also mentioned as draining energy. Particularly, when colleagues complain, gossip or do not listen to one another. One nurse illustrated this as follows:

“*Colleagues who complain [cost me energy]. […] They come to me looking for confirmation, but I do not want to be involved in these things.*”—nurse 40

Third, some nurses indicated that interactions with doctors can drain energy. This, for instance, occurs when nurses perceive doctors as inaccessible (nurses 12 and 41), when they experience a hierarchy between doctors and themselves (nurse 31), or when doctors fail to meet their promises to nurses (nurses 3 and 14).

### The threat to adopting a strict task approach

Another pattern that appeared from the data was that nurses who were structurally negative about their employability also generally had a strong focus on completing their required tasks such as regularly attending patients, checking their medication, or cleaning their wounds. Compared to nurses who were structurally positive about their employability, this group of nurses emphasize performing their tasks and addressed many aspects that hinder this and cost them energy. For instance, nurses mentioned losing energy when they are interrupted by phone calls (nurse 21), when physicians take too long to attend patients (nurse 28), and also when having to work on a special covid-19-shift (nurse 33). One nurse also explained being hindered by time pressures in her clinical tasks:

*“Everything should be done fast. Things should be quickly squeezed in between other tasks. I am often distracted when I work on my own task, then I have to regain my focus”*—nurse 18

The data also show that it costs these nurses energy when basic work conditions are not met, and this may affect their perceived employability. For instance, nurses are dissatisfied if information about patients is incomplete (nurse 39), stocks are not replenished (nurse 3) or medical devices are not working (nurse 29). Here, the impact of the covid-19 pandemic on meeting basic working conditions was also referred to. As with being disturbed, not satisfying these basic conditions drains and hinders this group of nurses from *doing their tasks*. As such, the strict task approach adopted by this group of nurses forms a threat to their employability.

### The opportunity to act professionally

The diaries illustrate that caring for patients, having professional autonomy to do so, and being able to develop professionally are very important for nurses. To start with, even nurses who are structurally negative about their employability find patient care important, and the nurses who are structurally positive about their employability gain considerable energy from elements relating to what is important in being a nurse. For this latter group of nurses, being disturbed in their tasks by unforeseen situations and urgent cases is stimulating. Above all, they are driven by a strong need to provide meaningful, wholesome care for patients. The following statements illustrate this:

*“[I am energised because] after quarantine, I am just happy to have meaning for patients again. To give them confidence, attention, explanations, etc.”*—nurse 39

*“[I am energised by] being meaningful for patients. [I am stimulated by] an intrinsic motivation to really contribute to the recovery of patients.”*—nurse 34

Nurses who are structurally positive about their employability also report being energized by having room for professional development within their jobs. They explicitly expressed being energized by learning new care techniques and procedures which help them to provide complex care to patients. For instance, nurses valued having time for in-depth development (nurses 10 and 44) and for working on cases that require complex care (nurses 21 and 26).

*“[I am stimulated by] my motivation to continue learning and developing. Today, a new situation occurred that stirred me and made me feel happy.”*—nurse 40

Finally, for this group of nurses, perceiving professional autonomy and control links to their perceptions of employability. They are energized by the feeling that they can do what, in their opinion, is best for their patients and also by having discretion in making decisions regarding the care plan. It is because of nurses’ extensive knowledge and experience that nurses feel they know best how to care for their patients.:

*“[I am stimulated because] even though the patient felt sick, I did not carry out all the actions the doctor recommended because I knew it would cause unnecessary suffering.”*—nurse 35

As with the need to provide wholesome care and for professional development, this need for autonomy lies at the heart of the nursing profession. The diary entries reflect that when nurses experienced either one of these three job characteristics during their shift, they were structurally more positive about their employability. As such, being able to “act professionally,” either by caring for patients, having room for professional development, or perceiving professional autonomy, provides opportunities for nurses’ employability.

## Discussion

This study has linked several job characteristics typical to the nursing profession to affect their employability. Also, this study has shown that the job characteristics that typify nurses’ shifts are dynamic over 3 weeks. Moreover, perceptions of employability also vary over time.

The diary approach resulted not only in myriad job characteristics but, more importantly, has illustrated that although nurses’ shifts can be typified by many job characteristics, only a few of the most salient ones are recalled when they reflect on their day. Unlike many studies on nurses’ job characteristics in which a set of job characteristics are quantitatively assessed (Broetje et al., 2020), this study asked nurses to reflect on the most salient aspects of their day. This more open approach illustrated how the dynamics in these job characteristics play a role in their experiences. Opening up to the dynamics in job characteristics enabled us to deepen and nuance our understanding of job characteristics.

We have shown that certain job characteristics seem to affect nurses’ employability. Guided by the JD-R model (Demerouti et al., 2001), our findings show which characteristics are demanding and resourceful to nurses. First, good social interactions seem to strengthen nurses’ perceived employability. Not only nurses who are structurally positive but also those who are structurally negative about their employability appear stimulated and energized by social interactions. Our findings are in line with other studies which show the importance of social interaction (Utriainen and Kyngäs, 2009; Broetje et al., 2020). Broetje et al. (2020) also refer to social interactions but name these interpersonal relations, and explain these are important for nurses as they form a hub between many different actors in patient care. Because of its potential positive effects, social interactions can be seen as job resources for nurses (Demerouti et al., 2001).

Second, nurses who are structurally negative about their employability often mentioned becoming drained when they were compromised in performing their tasks. Nurses who are structurally positive about their employability do not seem to be hindered by this. Those who were drained seemed to focus primarily on performing their required tasks. In their study, Broetje et al. (2020) also report that nurses find it important to do their work and that this energizes them. In contrast, our study shows that nurses with a strict task approach can be easily compromised in performing these tasks, and that this costs them energy. Comparing these findings with the JD-R model shows similarities with the category of hindering job demands (Lesener et al., 2019). The nurses who felt compromised in performing their tasks often had a structurally negative perception of their employability. As such, having a strict task approach can lower a nurse’s perceived employability.

Third, satisfying the cluster of elements that lay at the heart of the nursing ethos (wholesome care provision, professional development and autonomy) can energize nurses. Important to notice is that not all three elements need to be satisfied at all times but that nurses feel that they can “act professionally” during their shifts by satisfying either one of these elements. It was the nurses who were structurally positive about their employability that especially mentioned (some of) these elements during their shifts. We can link this to the literature on professionalism which highlights that professional employees have considerable professional autonomy, highly specialized knowledge and a strong service ethic (Noordegraaf, 2015). The needs expressed by several nurses in this study are very similar to these professional characteristics. Whereas the professionalism literature relates these professional characteristics to *classic* professionals such as physicians (Noordegraaf, 2015), this study shows that they also characterize *newer* professionals such as nurses (Noordegraaf, 2020). Future research could usefully look into the differences between the needs of classic and newer professionals.

Whereas Broetje et al. (2020) stressed the important role of supervisory support as a key job resource for nurses, the nurses in our study rarely mentioned either positive or negative aspects related to their line managers. This finding also contrasts with much of the Human Resource Management (HRM) literature where line managers are argued to fulfill an important role in supporting employees (Purcell and Hutchinson, 2007; Knies et al., 2020). An explanation might be found in the way line managers in medical settings are trained and selected. Nurses’ line managers have often themselves been trained as nurses. As a consequence, they may lack the managerial competencies required to invest in the employability of nurses and might fail to actively support nurses in this regard. This has similarly been argued as an important reason why medical managers of physicians do not invest in the employability of physicians in their teams (Van Leeuwen et al., 2022). It could be that nurses’ line managers have a somewhat passive role, and therefore that nurses do not recall them as being significant to their work.

Lastly, one of the most striking but unexpected findings of this study is that alongside the dynamics in job characteristics, nurses’ perceptions of their employability can also change over a few days. The data highlighted that several nurses felt differently about their employability on different days. The dynamics found in employability, which has also been seen in a study among physicians (Van Leeuwen et al., 2021a), sheds new light on this concept. Most research to date has treated employability as a static concept and measured it at only one point in time. Our findings provide evidence that cross-sectional study designs are biased and, therefore, not preferred to study relationships like this. By showing that employability is dynamic, this study indicates that it requires a longitudinal measurement to capture it.

### Study implications

This study contributes to the literature on nurses’ work experiences and employability. To understand this relationship well, it is important to acknowledge the daily dynamics in nurses’ job characteristics and perceptions of their employability. Consequently, future studies should use short-term longitudinal study designs to capture nurses’ work experiences and perceived employability. Additionally, it would be valuable if future studies conducted a content analysis in addition to our thematic analysis. This may reveal word patterns, word frequencies and other discourses of communication that relate to employability. Content analysis could help define the dynamic relationship between job characteristics and employability.

Further, this study is valuable for practice in that it can inform healthcare practitioners developing about targeted workplace interventions. This study shows that it is important for such interventions to focus on aspects that are important for nurses’ employability, such as ensuring time for social interactions and the professional elements seen as important by the nursing profession. Investing in these aspects could enhance nurses’ employability and reduce turnover. It is important, however, that interventions match the daily dynamics in both job characteristics and employability. As this study shows that nurses only recall the most prominent job characteristics, which vary between shifts, and that it is more important to address clusters of job characteristics instead of stand-alone job characteristics, interventions need to incorporate room for customization.

Moreover, this study has implications for line management in the nursing context by showing that line managers can become invisible to nurses in day-to-day practice. This is a concern since many studies show that line managers play an important role in achieving positive employee outcomes such as enhanced employability. Therefore, we would encourage healthcare practitioners to explore nurses’ needs for guidance and supervision and to train line managers’ people management skills accordingly. Additionally, we suggest making line managers more visible, for instance, by giving them a significant role in workplace interventions.

### Limitations

This study has some limitations. First, although we showed that experiences of both demanding and resource-enhancing aspects of work change within individuals over a short period, we could not draw conclusions on the long-term variation of the presumably dynamic job characteristics. Also, we did not examine why these work aspects changed over time. It would be valuable if future studies were to use longitudinal qualitative designs to further understand the mechanisms behind this variability as this could inform HR advisors and line managers how to create a work environment that is in line with nurses’ needs and preferences.

In addition to this limitation, we also found that nurses’ perceptions of their employability changed over a short period. While we were able to point at these unexpected dynamics, the data was not sufficient to provide insight into why the perceptions of employability changed on a within-person level. Because it may have serious implications for both research and practice, we recommend future research to study the daily dynamics in the perceptions of employability to find explanations for it.

Second, while a diary study is highly valuable in understanding the day-to-day experiences of participants, some dropout is inevitable and not all nurses completed a full set of diary entries. However, as Ohly et al. (2010) explain, organizational research findings are less affected by dropout than research based on counting occurrences. As we were not focused on counts, but seeking to collect as many diary entries as possible to identify potential patterns, we believe the dropouts will only minimally affect our data.

Third, while we benefitted from the longitudinal diary approach, the qualitative focus on the nurses of only two Dutch hospitals resulted in a limited understanding of whether these findings resonate with the whole nursing population. For generalization purposes, it would be relevant if future studies could examine the job characteristics and employability of nurses working in long-term care and primary care.

## Conclusion

This study examines how dynamic job characteristics relate to nurses’ employability. On a within level, the results show that both job characteristics *and* perceptions of employability change day to day. Between nurses, outcomes show that social interactions and the cluster of elements to “act professionally” (i.e., meaningful patient care, autonomy, and professional development) strengthen nurses’ employability, while too much focus on finishing tasks within the work hours threatens these perceptions. Although this study distinguished three categories of job characteristics relating to different perceptions of employability, to fully understand how these job characteristics affect nurses’ employability one has to capture the short-term dynamics that are present in a nursing context.

## Data availability statement

The raw data will not be available to protect anonymity of the respondents. Anonymized data is available upon request.

## Ethics statement

The studies involving human participants were reviewed and approved by Facultaire Ethische Toetsingscommissie (FETC) of the Faculty Law, Economics and Governance (LEG). Post address: Janskerkhof 3, 3512 BK Utrecht. The patients/participants provided their written informed consent to participate in this study.

## Author contributions

RV and EL conceptualized the research project, identified participants, analyzed the data, and drafted the manuscript. RV adjusted the application to fit nurses’ work. All authors contributed to the article and approved the submitted version.

## Conflict of interest

The authors declare that the research was conducted in the absence of any commercial or financial relationships that could be construed as a potential conflict of interest.

## Publisher’s note

All claims expressed in this article are solely those of the authors and do not necessarily represent those of their affiliated organizations, or those of the publisher, the editors and the reviewers. Any product that may be evaluated in this article, or claim that may be made by its manufacturer, is not guaranteed or endorsed by the publisher.
