# Supplementary material for: Capturing dynamics in nursing: a diary study of nurses’ job characteristics and ability and willingness to continue working
Source: Front Psychol. 2023 Jul 31;14:1112530. doi: 10.3389/fpsyg.2023.1112530 (PMC10423898; doi:10.3389/fpsyg.2023.1112530)
Supplement: Supplementary file 1 [file Table_1.docx]

**Appendix 1.** Example of the DearScholar application.

For more impressions on the layout of the application, visit the DearScholar Github (Reproduced with permission from Kruyen, 2023) [Kruyen,P.M(2023). [github url]]

**Description Screen example in app**


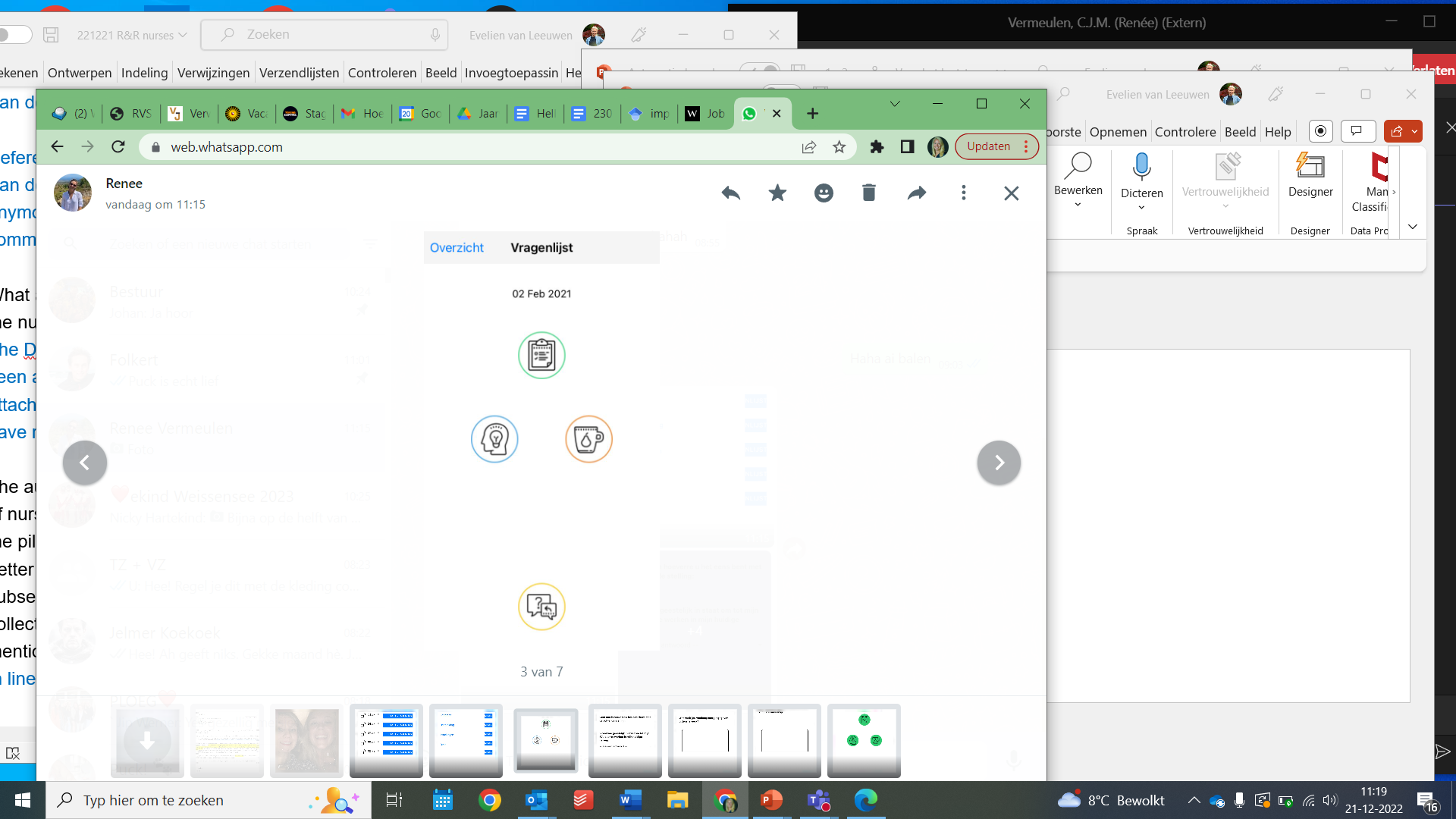

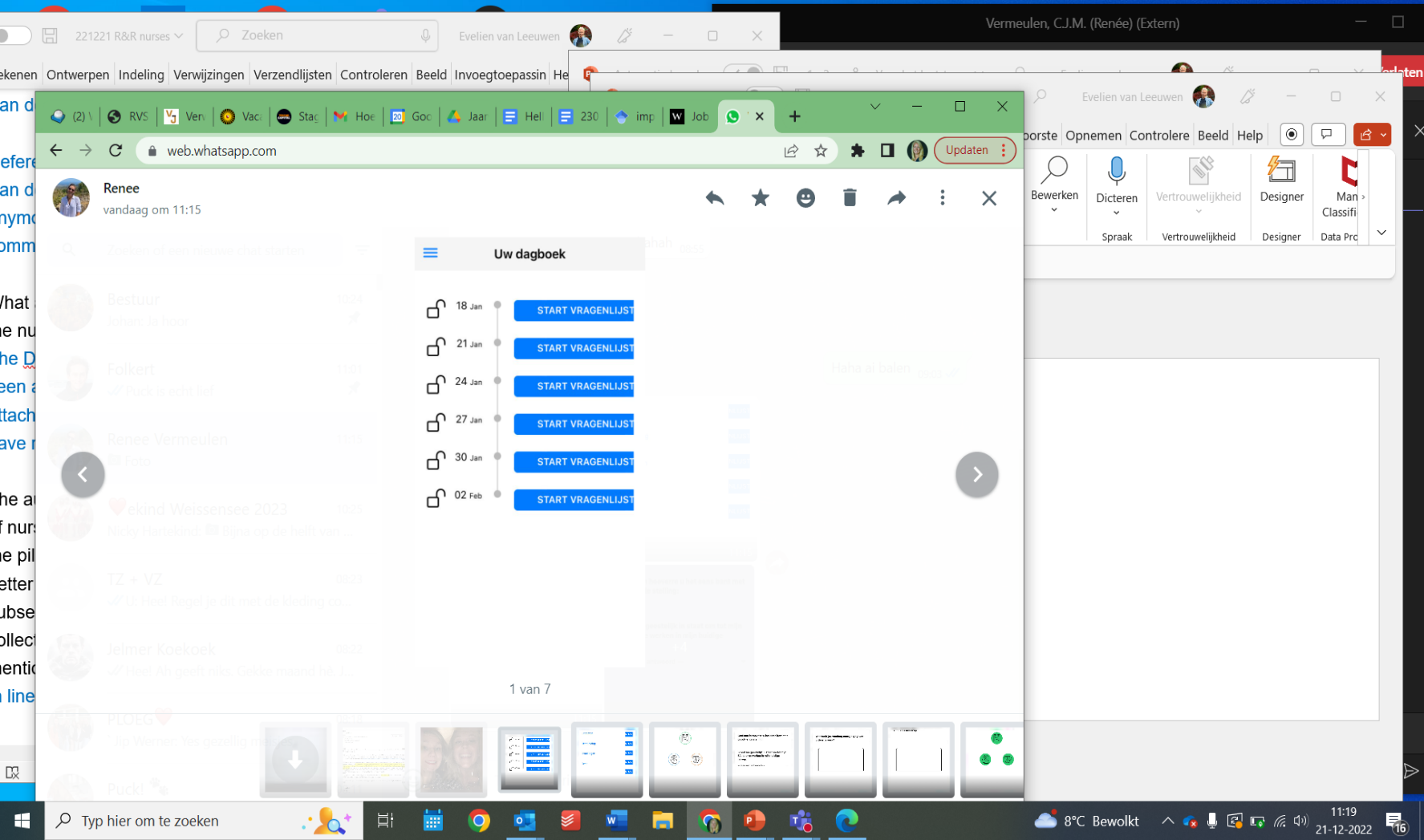


This is opening screen when a nurse starts the survey. The questions that are part of the survey appear when they push on these buttons.

This is the start screen that nurses see when opening the application. On the left the data appear on which nurses complete the diary. On the right they can find the buttons to open the accompanying survey.


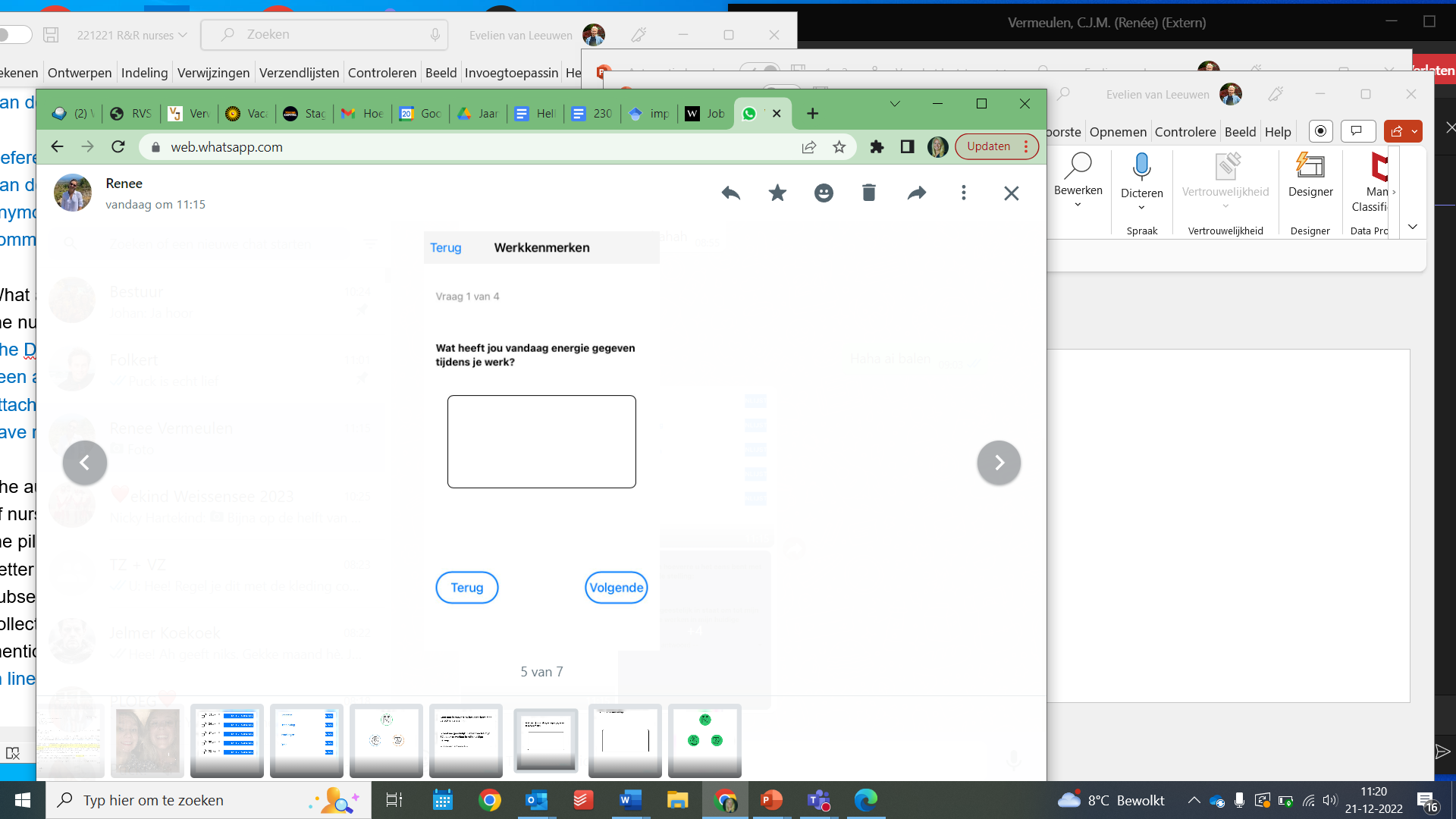

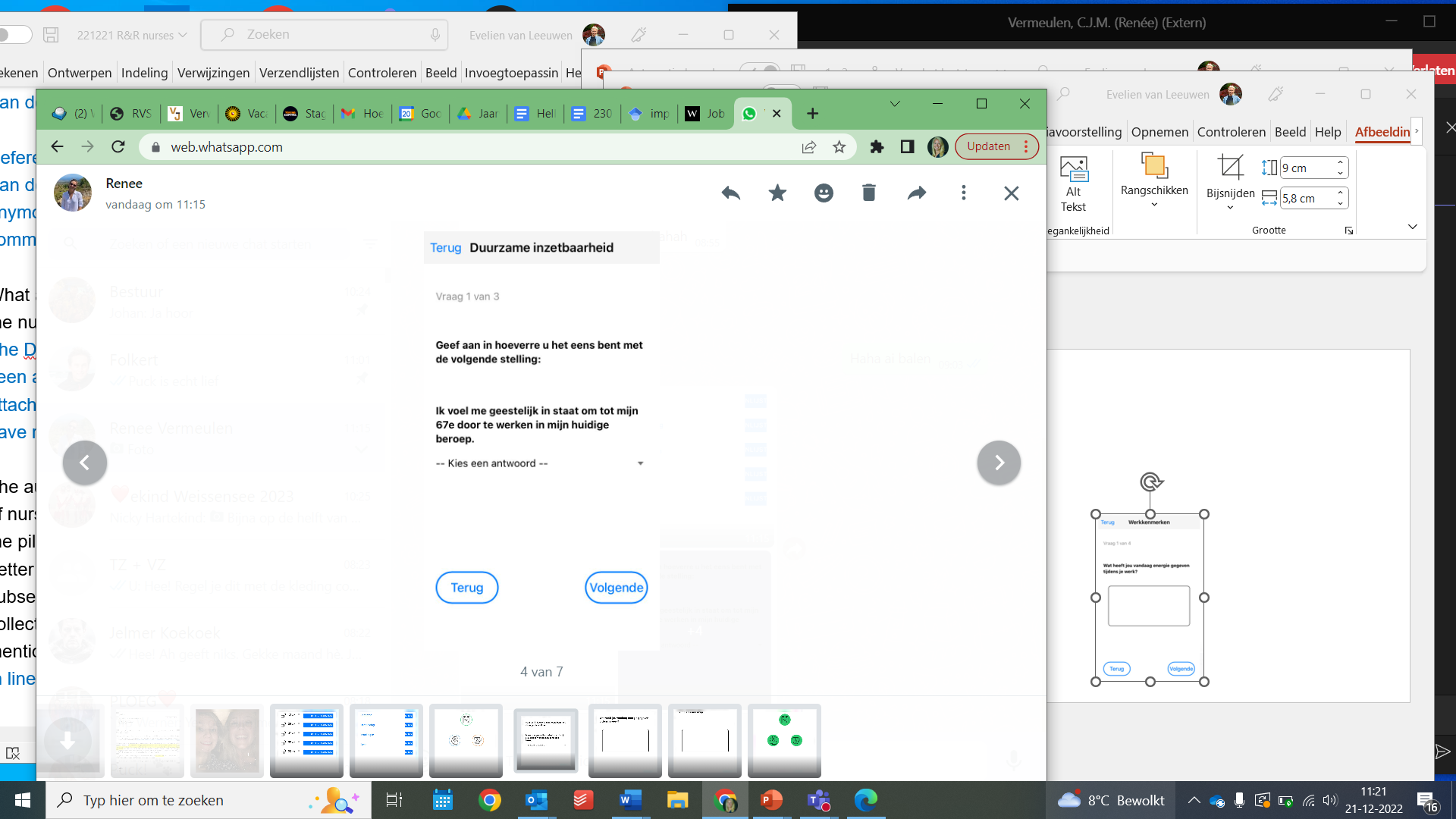


This screen shows an example item used to measure employability. It asks to what extent nurses agree with the statement: “I feel mentally able to remain employed in nursing until the retirement age.”

This screen shows an example item asking nurses what gave this nurse energy during the shift. At the top you can see that this is 1 question out of 4 questions in this category and at the bottom you find the “back” and “next” buttons.

**Appendix 2.** Overview of the participating nurses

| **Nurse** | **Hospital** | **Age category** | **Educational level** | **Tenure** |
| --- | --- | --- | --- | --- |
| **1** | 2 | 60+ | Undergraduate | > 40 years |
| **2** | 2 | 20-29 | Undergraduate | 0-5 years |
| **3** | 2 | 30-39 | Undergraduate | 11-15 years |
| **4** | 2 | 20-29 | Undergraduate | 6-10 years |
| **5** | 2 | 30-39 | High school (A-levels) | 6-10 years |
| **6** | 2 | 60+ | High school (A-levels) | > 40 years |
| **7** | 2 | 50-59 | Undergraduate | 31-35 years |
| **8** | 2 | 50-59 | Graduate | 31-35 years |
| **9** | 2 | 30-39 | High school (A-levels) | 6-10 years |
| **10** | 2 | 30-39 | Undergraduate | 6-10 years |
| **11** | 2 | 30-39 | Undergraduate | 11-15 years |
| **12** | 2 | 40-49 | High school (A-levels) | 21-25 years |
| **13** | 2 | 20-29 | Undergraduate | 5-10 years |
| **14** | 2 | 20-29 | Undergraduate | 0-5 years |
| **15** | 2 | 40-49 | Undergraduate | 21-25 years |
| **16** | 2 | 50-59 | Undergraduate | 21-25 years |
| **17** | 2 | 50-59 | High school (A-levels) | > 40 years |
| **18** | 2 | 30-39 | Undergraduate | 6-10 years |
| **19** | 2 | 50-59 | Undergraduate | 26-30 years |
| **20** | 2 | 40-49 | High school (A-levels) | 21-25 years |
| **21** | 2 | 40-49 | Undergraduate | 21-25 years |
| **22** | 2 | 30-39 | Undergraduate | 6-10 years |
| **23** | 1 | 30-39 | Graduate | 0-5 years |
| **24** | 1 | 40-49 | Undergraduate | 16-20 years |
| **25** | 1 | 40-49 | Undergraduate | 21-25 years |
| **26** | 1 | 20-29 | Graduate | 0-5 years |
| **27** | 1 | 20-29 | Undergraduate | 6-10 years |
| **28** | 1 | 60+ | High school (A-levels) | > 40 years |
| **29** | 1 | 20-29 | Undergraduate | 0-5 years |
| **30** | 1 | 50-59 | High school (A-levels) | 31-35 years |
| **31** | 1 | 20-29 | Undergraduate | 6-10 years |
| **32** | 1 | missing | missing | Missing |
| **33** | 1 | 60+ | Graduate | > 40 years |
| **34** | 1 | 40-49 | High school (A-levels) | 26-30 years |
| **35** | 1 | 20-29 | Undergraduate | 0-5 years |
| **36** | 1 | 30-39 | Undergraduate | 16-20 years |
| **37** | 1 | 20-29 | Undergraduate | 0-5 years |
| **38** | 1 | 40-49 | Undergraduate | 16-20 years |
| **39** | 1 | 30-39 | Undergraduate | 11-15 years |
| **40** | 1 | 30-39 | High school (A-levels) | 6-10 years |
| **41** | 1 | 20-29 | Undergraduate | 0-5 years |
| **42** | 1 | 20-29 | Undergraduate | 0-5 years |
| **43** | 1 | 30-39 | Undergraduate | 6-10 years |
| **44** | 1 | 40-49 | High school (A-levels) | 0-5 years |
| **45** | 1 | 50-59 | High school (A-levels) | 31-35 years |
| **46** | 1 | 40-49 | Graduate | 21-25 years |

**Appendix 3.** Items used in the intake survey and in the DearScholar app.

| **Demographics** | |
| --- | --- |
| What is your age? | *M*=40.10 (*SD*=11.47)  Min=25  Max=64 |
| What is your gender? | Female (*n*=44)  Male (*n*=2)  Non-binary (*n*=0)  Prefer not to say (*n*=0) |
| What is your highest level of education? | Primary education (*n*=0)  Vocational education (*n*=0)  Higher education (*n*=12)  Bachelor’s degree (*n*=29)  Master’s or doctoral degree (*n*=6)  Unknown (*n*=1) |
| Have you received specialist education? *(If yes, name all these educations)* | Yes (*n*=33)   - Oncology (*n*=12) - Paediatrics (*n*=10) - Intensive Care (*n*=5) - Neurology; nurse specialist (*n*=3) - Haematology; emergency room nursing; medium care; practical trainer (*n*=2) - Wound care; neonatology; lactation science; palliative care; nursing ethics; recovery nursing; ostomy care; gynaecology; obstetrics; evidence based practice (*n*=1)   No (*n*=12)  Unknown (*n*=1) |
| How many years have you been working as a nurse? | *M*=17.47 (*SD*=12.81)  Min=1.50  Max=44.00 |
| **Workplace** | |
| What hospital do you work for? | Hospital 1 (academic hospital) (*n*=24)  Hospital 2 (general hospital) (*n*=22) |
| What is your functional title? | *Open question* |
| In what division do you work? | *Open question* |
| **Employment conditions** | |
| How are you employed? | Paid employment (*n*=41)  Temporary employment (*n*=2)  Self-employed (*n*=1)  Differently (*n*=1)  Unknown (*n*=1) |
| How many hours are you contractually employed each week? | *M*=29.10 (*SD*=6.54)  Min=0  Max=40.00 |
| How many hours do you actually work? | *M*=29.94 (*SD*=6.25)  Min=8.00  Max=40.00 |

**Appendix 4.** All themes and subthemes used in the coding process.

| **Theme** | **Sub-theme** | **Example quote** |
| --- | --- | --- |
| Covid-19 | Covid-19 regulations | *Subtheme: ways of working during Covid-19*  “It costs energy to work in special shifts of patients in covid-19 isolation, 8 hours in special suits, with only two breaks” |
|  | Reorganization of patients |  |
|  | Way of working during Covid-19 |  |
| Employability | Stable |  |
|  | Dynamic |  |
| Hygiene factors in the job | Threshold to come to work | *Subtheme: secondary work arrangements*  “Recently, the hospital started offering yoga and meditation classes – yoga gives me energy and I like that this is offered” |
|  | Lunchtime |  |
|  | Breaks |  |
|  | Salary |  |
|  | Secondary work arrangements |  |
|  | Other work arrangements |  |
| Management | Management decides work content | *Subtheme: management does not listen to employees*  “It hinders me that policy makers within the hospital do not listen to nurses and think they can do whatever they feel like. They do not cater to nurses’ needs” |
|  | Management does not listen to employees |  |
|  | Management does not self-reflect |  |
|  | Management puts efficiency above employees’ safety |  |
| Interactions with specialists | Social interaction | *Subtheme: work-related interactions*  “It costed me energy to wait for the physician – while pressing a bleeding wound. The physician told me that they were ‘right back’, but still weren’t back 15 minutes later” |
|  | Work-related interaction |  |
| Interactions with colleagues | Climate at the department | *Subtheme: social interaction*  “What gave me energy today were the pleasant and good conversations with my colleagues” |
|  | Social interaction |  |
|  | Work-related interaction |  |
| Interactions with patients’ family | Emotional interaction | *Subtheme: emotional interaction*  “The gratitude of a patients’ mother, who will return home tomorrow, but only with a uncertain future for her child” |
|  | Care interaction |  |
| Interactions with patients | Emotional interaction | *Subtheme: care interaction*  “It gave me energy that I was of help to a patient to make the pain more bearable” |
|  | Social interaction |  |
|  | Care interaction |  |
| Interactions with students | Social interaction | *Subtheme: work-related interaction*  “What stimulated me today was the challenge of coaching my students well. One of them underestimates everything, the other one runs too fast” |
|  | Work-related interaction |  |
| Interactions with team managers | Social interaction | *Subtheme: work-related interaction*  “It gave me energy that there was room for my conversation with my team manager. This does not happen very often” |
|  | Work-related interaction |  |
| Organization behind the job | Administration | *Subtheme: efficiency of procedures*  “What cost me energy is the bureaucratical non-sense of being sent from pillar to post concerning a project” |
|  | Efficiency of procedures |  |
|  | Equipment |  |
|  | Work schedules and staffing |  |
|  | Support staff |  |
|  | Unexpected changes during the day |  |
|  | Working from home |  |
|  | Controls, checks, protocols |  |
|  | Changes in the work space |  |
|  | Changes in the required care |  |
| Personal features | Personality | *Subtheme: personal state or health is deprived*  “Today I was tired because I did not sleep well during the day {in preparation for the night shift}” |
|  | External factors for nurses |  |
|  | Personal state or health is deprived |  |
| Professional development | Autonomy | *Subtheme: learning & development*  “It gave me energy to brainstorm with colleagues about how we can develop ourselves as an department” |
|  | Learning & development |  |
| Tasks | Administration | *Subtheme: to do list*  “It stimulated me to finish a task that had been on my to do list for a long time” |
|  | Variety in tasks |  |
|  | Research |  |
|  | Training new colleagues |  |
|  | Training students |  |
|  | Organizational tasks |  |
|  | Red tape |  |
|  | To do list |  |
|  | Care task |  |
| Executing care | About doing the care tasks | *Subtheme: about feeling whilst caring*  “It gave me energy to see one of my patients getting better and to see him being discharged today” |
|  | About feeling whilst caring |  |
| Work pressure | Making work-pressure manageable | *Subtheme: directly care-related work-pressure*  “The work-pressure cost me energy. I have to prioritize continuously as many people ask for your attention at the very same time” |
|  | Directly care-related work-pressure |  |
|  | Good work pressure |  |
|  | Too high work pressure |  |
|  | Too low work pressure |  |

References

Kruyen, P.M. (Kruyen, 2023). DearScholar: An open-source smartphone app for longitudinal scientific qualitative and quantitative (self-report) diary, log and survey research. Github. https://github.com/pmkruyen/dearscholar
